# Genome-wide identification, characterization, and evolutionary analysis of flowering genes in radish (*Raphanus sativus* L.)

**DOI:** 10.1186/s12864-017-4377-z

**Published:** 2017-12-19

**Authors:** Jinglei Wang, Yang Qiu, Feng Cheng, Xiaohua Chen, Xiaohui Zhang, Haiping Wang, Jiangping Song, Mengmeng Duan, Haohui Yang, Xixiang Li

**Affiliations:** Institute of Vegetables and Flowers, Chinese Academy of Agricultural Sciences, Key Laboratory of Biology and Genetic Improvement of Horticultural Crops, Ministry of Agriculture, Beijing, 100081 China

**Keywords:** *Raphanus sativus* L., Genome-wide, Flowering genes, Regulatory pathway networks, Evolution

## Abstract

**Background:**

Radish (*Raphanus sativus* L.) belongs to the family Brassicaceae, and is an economically important root crop grown worldwide. Flowering is necessary for plant propagation, but it is also an important agronomic trait influencing *R. sativus* fleshy taproot yield and quality in the case of an imbalance between vegetative and reproductive growth. There is currently a lack of detailed information regarding the pathways regulating the flowering genes or their evolution in *R. sativus*. The release of the *R. sativus* genome sequence provides an opportunity to identify and characterize the flowering genes using a comparative genomics approach.

**Results:**

We identified 254 *R. sativus* flowering genes based on sequence similarities and analyses of syntenic regions. The genes were unevenly distributed on the various chromosomes. Furthermore, we discovered the existence of *R. sativus* core function genes in the flowering regulatory network, which revealed that basic flowering pathways are relatively conserved between *Arabidopsis thaliana* and *R. sativus*. Additional comparisons with *Brassica oleracea* and *Brassica rapa* indicated that the retained flowering genes differed among species after genome triplication events. The *R. sativus* flowering genes were preferentially retained, especially those associated with gibberellin signaling and metabolism. Moreover, analyses of selection pressures suggested that the genes in vernalization and autonomous pathways were more variable than the genes in other *R. sativus* flowering pathways.

**Conclusions:**

Our results revealed that the core flowering genes are conserved between *R. sativus* and *A. thaliana* to a certain extent. Moreover, the copy number variation and functional differentiation of the homologous genes in *R. sativus* increased the complexity of the flowering regulatory networks after genome polyploidization. Our study provides an integrated framework for the *R. sativus* flowering pathways and insights into the evolutionary relationships between *R. sativus* flowering genes and the genes from *A. thaliana* and close relatives.

**Electronic supplementary material:**

The online version of this article (10.1186/s12864-017-4377-z) contains supplementary material, which is available to authorized users.

## Background

Flowering is a necessary part of plant propagation, and the process from bolting to blooming is a crucial period for the transition of Brassicaceae plants from vegetative to reproductive growth. Comprehensively characterizing the regulatory mechanisms underlying bolting and blooming may enable researchers to influence the balance between vegetative and reproductive growth, which may ultimately affect the yield and quality of Brassicaceae crops.

Approximately 174 genes are believed to regulate flowering in the model plant *Arabidopsis thaliana*, which are involved in six major pathways [i.e., vernalization, photoperiod and circadian clock, ambient temperature, gibberellin (GA), age, and autonomous pathways] influencing the bolting or blooming process [1]. Although different genes are responsible for different internal and environmentally mediated flowering pathways, the different pathways appear coordinated primarily by a few floral integrator genes, including *FLOWERING LOCUS T* (*FT*), *LEAFY* (*LFY*), and *SUPPRESSOR OF OVEREXPRESSION OF CONSTANS1* (*SOC1*) [1]. The recent completion of genome sequences and the development of novel computational analysis techniques have enabled the genome-wide identification and characterization of flowering genes in economically important plants. For example, 900 and 275 putative flowering genes in *Triticum aestivum* and *Hordeum vulgare* [2] respectively, 96, 98 and 304 flowering gene homologs in *Lotus corniculatus* var. *japonicus*, *Medicago truncatula* and *Glycine max* [3] separately, have been identified. The genes regulating bolting and flowering vary among different crops.

*Raphanus sativus* is a member of the family Brassicaceae, and is cultivated worldwide. It has recently undergone tetraploidization events (α and β) with *A. thaliana*, *B. oleracea* and *B. rapa*, as well as a whole genome triplication with *B. oleracea* and *B. rapa* following their divergence from *A. thaliana* [4, 5]. *R. sativus* has similar flowering habits to *A. thaliana*, *B. oleracea*, and *B. rapa*, with a highly variable flowering time and diverse responses to temperature and/or day length. Fifty flowering miRNAs targeting 154 transcripts [6], and 95 flowering genes differentially expressed between the vegetative and reproductive stages [7], have been identified in *R. sativus*. Additionally, 290 flowering genes have been detected in the *R. sativus* genome [8]. However, little attention has been paid to their characteristics or evolution of the genes in different pathways regulating *R. sativus* flowering.

In this study, we systematically identified flowering genes in the *R. sativus* genome, and uncovered new details regarding the presence or absence of these genes. We also investigated dominant pathways as well as the evolutionary relationships and expression profiles among the flowering genes. Our findings may provide useful information and enlightenment for breeders to improve bolting and flowering in *R. sativus* and other Brassicaceae crops.

## Methods

### Data resources

Details regarding the annotated *A. thaliana* genome were downloaded from the TAIR10 website (http://www.arabidopsis.org) [9]. The *R. sativus, B. rapa* and *B. oleracea* genome assembly and gene annotation data were downloaded from the BRAD database (http://brassicadb.org/brad/) [10]. Genomic data for *Vitis vinifera*, *Populus trichocarpa*, *Carica papaya*, and *Thellungiella salsuginea* were obtained from the Genoscope (http://www.genoscope.cns.fr/spip/), JGI Genome Portal (http://genome.jgi.doe.gov/), plantGDB (http://www.plantgdb.org/), and Omicslab (http://omicslab.genetics.ac.cn/resources.php) databases, respectively. *R. sativus* RNA-seq data [11] are available at EMBL/NCBI/SRA (PRJNA413464).The core eukaryotic genes were downloaded from CEGMA (http://korflab.ucdavis.edu/datasets/cegma/) [12].

### Identification of flowering gene homologs in *Raphanus sativus*

We identified homologous genes using a combination of similarity- and synteny-based approaches. In the similarity-based approach, BLASTP searches were conducted against *R. sativus* protein sequences using the following conditions: E-value <1e-20, identity >50%, coverage >60%, and match length > 60 amino acids. In the synteny-based approach, SynOrths software (http://brassicadb.org/brad/downloadOverview.php), which determines whether two genes are a conserved syntenic pair based on sequence similarities and homologies of their flanking genes, was used to identify syntenic *A. thaliana* and *R. sativus* genes [13]. We further defined homologous relationships among the similar and syntenic genes. Multiple gene sequences were aligned using CLUSTALW [14], and phylogenetic trees were constructed using the neighbor-joining method of the MEGA 6.0 software (1000 bootstrap replicates) [15]. Putative homologous genes were manually checked on the phylogenetic trees.

### Localization of flowering genes in the *Raphanus sativus* genome

To construct physical maps indicating the distribution of flowering genes, genome localization details for the predicted *R. sativus* flowering genes were collected from the annotation information. The MG2C (http://mg2c.iask.in/mg2c_v2.0/) program was used to visualize the putative flowering genes on nine pseudo-molecular chromosomes [16].

### Flowering gene expression analysis based on RNA-seq data

We analyzed the transcriptomes of six different tissues (i.e., flowers, siliques, leaves, stem, callus, and roots) collected from *R. sativus* inbred line XYB36–2 [11]. Transcript abundance was calculated according to the FPKM method (fragments per kilobase of exon per million mapped reads) using Cufflinks [17] and TopHat2 [18]. Heatmaps were generated with the R package pheatmap [19].

### Non-synonymous/synonymous substitution ratios of flowering gene pairs between *Arabidopsis thaliana* and *Raphanus sativus*

The non-synonymous/synonymous substitution ratio (Ka/Ks) of homologous gene pairs is related to the evolutionary selection patterns of the corresponding genome. In the calculation of Ka/Ks, The full length of amino acid sequences of the *R. sativus* and *A. thaliana* flowering genes underwent pairwise alignments using MUSCLE [20] firstly. Then, The aligned amino acid sequences were translated into the corresponding nucleotides coding sequences using PERL scripts. Finally, the translated nucleotides coding sequences were used as input files in computing Ka/Ks values using Li-Wu-Luo model [21] integrated in KaKs_Calculator2 software [22]. All variable sites of the alignment pairs were used in the Ka/Ks calculation. To detect selection pressures, Ka/Ks ratios greater than 1, less than 1, and equal to 1 were considered to represent positive selection, negative or stabilizing selection, and neutral selection, respectively.

## Results

### Identification of *Raphanus sativus* flowering genes

There are 174 genes, including 24 μ-RNA genes, with known functions affecting *A. thaliana* flowering time [1]. We focused on the 160 protein-coding genes to identify homologous *R. sativus* flowering genes. We identified 254 *R. sativus* flowering genes (Additional file 1), and determined that most of the *A. thaliana* flowering genes have putative *R. sativus* homologs (139 out of 160). Homologs in the *R. sativus* genome were lacking for 21 genes, and most of these genes (15 of 21) have functionally redundant effects on flowering (Additional file 2). Interestingly, all of the lost genes (8 genes) which belonging to photoperiod pathway, circadian clock, and light signaling genes set have function redundant genes retained in *R. sativus.*

### Distribution of *Raphanus sativus* flowering genes on pseudo-molecular chromosomes

**Fig. 1:**
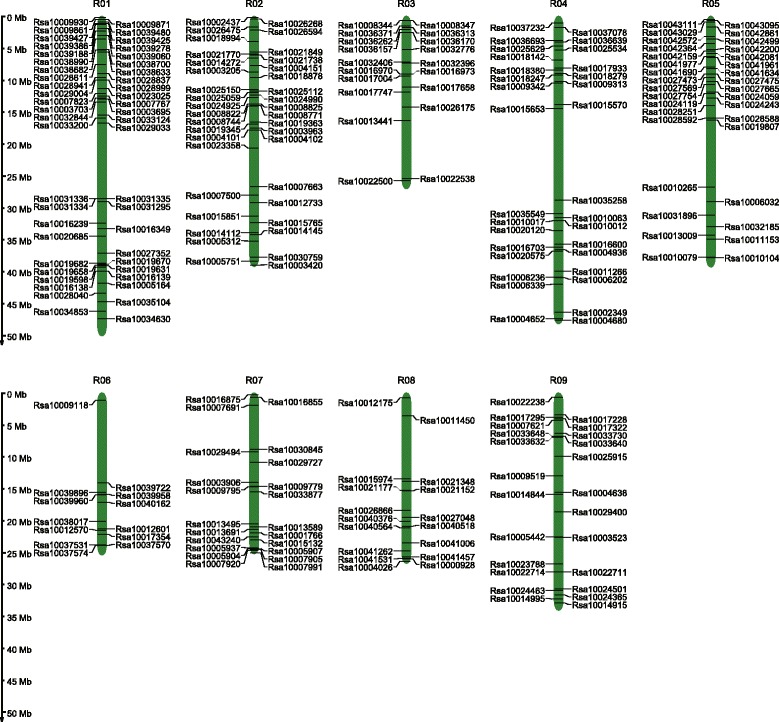
Distribution of flowering genes on *Raphanus sativus* chromosomes. Green bars represent pseudo-chromosomes. The black line on olive bars indicates the location of flowering genes on pseudo-chromosomes. Values corresponding to the scales on the black vertical line indicate physical distance

We mapped 247 *R. sativus* flowering genes onto pseudo-molecular chromosomes, while the remaining seven genes were assigned to unanchored scaffolds (Fig. 1). The distribution of these genes was uneven, with 48 genes localized on chromosome 1, representing 19.43% of the flowering genes. Only 14 flowering genes (5.66%) were detected on chromosome 6, with most located on the bottom half.

### Comparison of flowering genes from *Arabidopsis thaliana*, *Brassica oleracea, Brassica rapa*, and *Raphanus sativus*

**Fig. 2:**
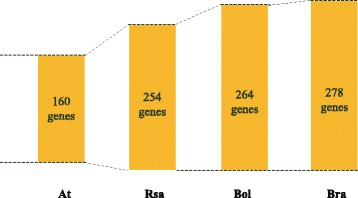
Flowering genes in different species. At, *Arabidopsis thaliana*; Rsa, *Raphanus sativus*; Bol, *Brassica oleracea*; Bra, *Brassica rapa*. Numbers in boxes correspond to the number of genes

As Brassicaceae species, *B. oleracea* and *B. rapa* have been sequenced and studied in-depth [23–25]. We used the abovementioned method to identify homologous flowering genes in *B. oleracea* and *B. rapa*. The fewest number of flowering genes were identified for *R. sativus* (Fig. 2 and Additional file 3), even though it had the second most annotated genes (43,240) [11], which is between *B. oleracea* (45,758) [23] and *B. rapa* (41,174) [24]. There were no significant differences in the number of identified flowering genes among *R. sativus*, *B. oleracea* and *B. rapa* (Chi-squared test = 2.3224, *P* value = 0.1275).

Except for four genes that could not be categorized, the putative *R. sativus*, *B. oleracea*, and *B. rapa* flowering genes were classified into the following four gene sets: GA signaling and metabolism; vernalization and autonomous pathways; photoperiod pathway, circadian clock, and light signaling; and meristem response and development, according to the classification of *A. thaliana* genes [1] (Table 1 and Additional file 3). Most of the genes belonged to the photoperiod pathway, circadian clock, and light signaling gene set. There was little difference in the numbers of *R. sativus*, *B. oleracea*, and *B. rapa* genes associated with GA signaling and metabolism*.*

**Table 1:** Number of flowering genes in *Arabidopsis thaliana*, *Raphanus sativus*, *Brassica oleracea*, and *Brassica rapa*

Flowering pathways and their gene sets	*A. thaliana*	*R. sativus*	*B. oleracea*	*B. rapa*
Photoperiod pathway, circadian clock, light signaling	67	101	107	114
Gibberellin signaling and metabolism	21	35	33	34
Vernalization and autonomous pathways	49	72	72	81
Meristem response and development	19	38	44	42
Other	4	8	8	7
Total	160	254	264	278

### Dominant pathways and key families of *Raphanus sativus* flowering genes

#### Photoperiod pathway, circadian clock, and light signaling

Plants can sense day length changes and use them to control the onset of flowering. We identified 101 *R. sativus* genes that were homologous to 58 *A. thaliana* genes of photoperiod pathway, circadian clock, and light signaling (Additional file 1).

*CONSTANS* (*CO*), which acts as a point of integration of the internal circadian clock and the external day-night cycles, plays a central role in photoperiodic flowering control of plants [26]. In *R. sativus*, one copy was identified to be homologous of *CO*. Significantly, the genes involved in circadian clock including *CCA1*, *LHY*, *TOC1*, *GI*, *CDF1* and *LKP2* [27–30] were identified in *R. sativus. LKP2* belongs to a family of F-box proteins, which also include *ZTL* and *FKF1* [27]. *ZTL* and *FKF1* are both lost and *LKP2* have three tightly linked copies in *R. sativus,* that are similar to that of *B. rapa* [25], suggesting the lose of *ZTL* and *FKF1* and the local triplication event of *LKP2* may have taken place in the common ancestor of *R. sativus* and *B. rapa*. In addition, *CRY1*, *CRY2, PHYA*, *PHYB*, *PHYC*, and *PHYE* being implicated in plant light signaling pathways [31] were identified in *R. sativus*.

#### Vernalization and autonomous pathways

Many plants growing in temperate climates require vernalization (i.e., prolonged exposure to low temperatures), which involves the silencing of *FLC*, to initiate or accelerate the flowering process [32]. Similar to the genes of the vernalization pathway, genes in the autonomous pathway normally indirectly promote flowering by repressing the floral repressor *FLC* [33]. *FLC*, which is a MADS-box gene, is the major flowering repressor in the vernalization pathway [34]. Three *FLC* homologs were identified in *R. sativus*. As expected, most of vernalization-response genes including *VIN3*, *VRN1*, *VRN2, FRI* were also identified in this study [35–38]. Furthermore, in the FLC-independent vernalization pathway, prolonged exposure to cold conditions can elevate *AGL19* and *AGL24* expression levels, which can activate *LFY* and *AP1* expression and eventually leads to flowering [39]. The *R. sativus* contained three copies of *AGL19* and two copies of *AGL24*.

Moreover, we also identified homologous genes in autonomous pathway, including *LD*, *FCA*, *FY*, *FPA*, *FVE*, *FLD*, and *FLK.* All autonomous genes are indirectly involved in inducing early flowering through the repression of *FLC* [40].

#### Gibberellin signaling and metabolism

The initiation of flowering in *A. thaliana* under non-inductive short-day conditions can be promoted by GA. There are 35 genes likely related to the GA pathway in *R. sativus.* The GA pathway genes are classified as those associated with GA biosynthesis (e.g., *GA2ox*, *GA3ox*, *GA20ox* [41]) and those acting as key signal transduction factors (e.g., *SLY1, RGA*, and *GID1* [42]). Except for *GAI*, homologs of the all GA biosynthesis genes and transduction factors were retained in *R. sativus*. The *GAI* and *RGA* genes are members of the DELLA family, which repress GA-induced vegetative growth and floral initiation [43].

#### Meristem response and development

The onset of flowering is largely dependent on the expression of a relatively small number of central floral pathway factors that integrate signals from several related pathways during floral transitions [44]. In our study, 38 *R. sativus* genes were identified as floral integrators or were associated with the flower meristem, including *SOC1*, *FT*, *AP1*, *LFY*, and *FD*. Jung et al. (2016) did not detect *R. sativus LFY* through transcriptomic analysis [45], while, we detect two copies, although they both little expressed*,* which indicated *LFY* may expressed in specific periods and tissues.

### Differential retention of flowering genes in various species

**Fig. 3:**
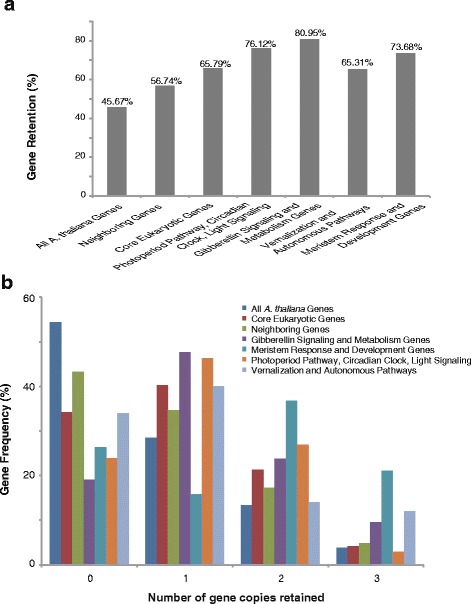
Number of flowering gene homologs retained as syntenic genes in *Raphanus sativus*. The retained homologs among the four gene sets of flowering genes and their immediate neighbors, all *A. thaliana* genes, and 459 core eukaryotic genes were included in the analysis. **a** Ratios of the retained genes in different gene sets. **b** Number of retained homologous genes in different gene sets

The gene dosage hypothesis predicts that genes whose products are dose-sensitive or interact with other proteins or in networks are over-retained , [46]. We compared the retention of genes from each of the above-mentioned four gene sets and three other gene sets: all *A. thaliana* genes, 2780 genes flanking the flowering genes (10 on either side), and 459 core eukaryotic genes. Overall, 80.95% of the GA signaling and metabolism genes, 76.12% of the photoperiod pathway, circadian clock, and light signaling genes, 73.68% of the meristem response and development genes, and 65.31% of the vernalization and autonomous pathway genes were retained as syntenic genes. In contrast, 65.79% of the core eukaryotic genes, 56.74% of the neighboring genes, and 45.67% of all *A. thaliana* genes were retained as syntenic genes (Chi-squared test = 232.5112, *P* < 0.001) (Fig. 3a). Most (57.89%) of the meristem response and development genes were retained as two or three copies (Fig. 3b).

### Selection pressure on flowering pathway gene sets

**Fig. 4:**
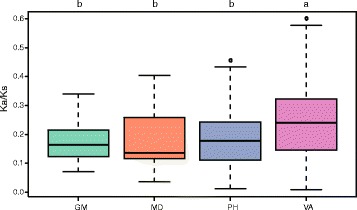
Direction and magnitude of natural selection acting on different flowering gene sets. Quantile boxplots (0.25, 0.75) show the distribution of Ka/Ks values for homologous gene pairs. The horizontal bar in each box indicates the median value. The upper and lower bars correspond to the upper and lower adjacent values 1.5-times outside the inter-quartile range. Outliers are plotted as discrete dots. Different letters indicate significantly different values (*P* < 0.01) as measured by Scheffé’s test. GM, gibberellin signaling and metabolism gene set; MD, meristem response and development gene set; PH, photoperiod pathway, circadian clock, and light signaling gene set; VA, vernalization and autonomous pathways gene set

The Ka/Ks ratios for homologous gene pairs were estimated to determine the direction and magnitude of natural selection acting on the *R. sativus* flowering genes. The mean Ka/Ks ratios of different flowering gene sets ranged from 0.18 to 0.25 (Fig. 4 and Additional file 4), suggesting that negative selection had acted against extreme polymorphic variants in flowering genes. In particular, genes of the vernalization and autonomous pathways appear to have been subjected to less negative selection pressures than the genes from other pathways.

### *Raphanus sativus* Flowering gene expression analysis

To characterize the divergence in the expression patterns of homologous genes and confirm their involvement in flowering, we analyzed the expression of the putative *R. sativus* flowering genes*.* By comparing transcript abundances in roots, stem, leaves, flowers, siliques, and callus, we determined that the expression of 16 putative flowering genes was undetectable in all tissues (Additional file 5). Furthermore, transcripts for most of the expressed genes (183 of 254) accumulated in flowers (Additional file 5), with seven genes that were preferentially or specifically expressed in flowers. Four of these seven genes (i.e., *LMI1*, *SPL4*, *SPL5*, and *TFL1*) were related to meristem response and development. Besides, it was found that *COP1* and *VIL1*, only have one copy in *R. sativus*and not expressed in all tissues. It seems that these genes have lost function in *R. sativus*.

**Fig. 5:**
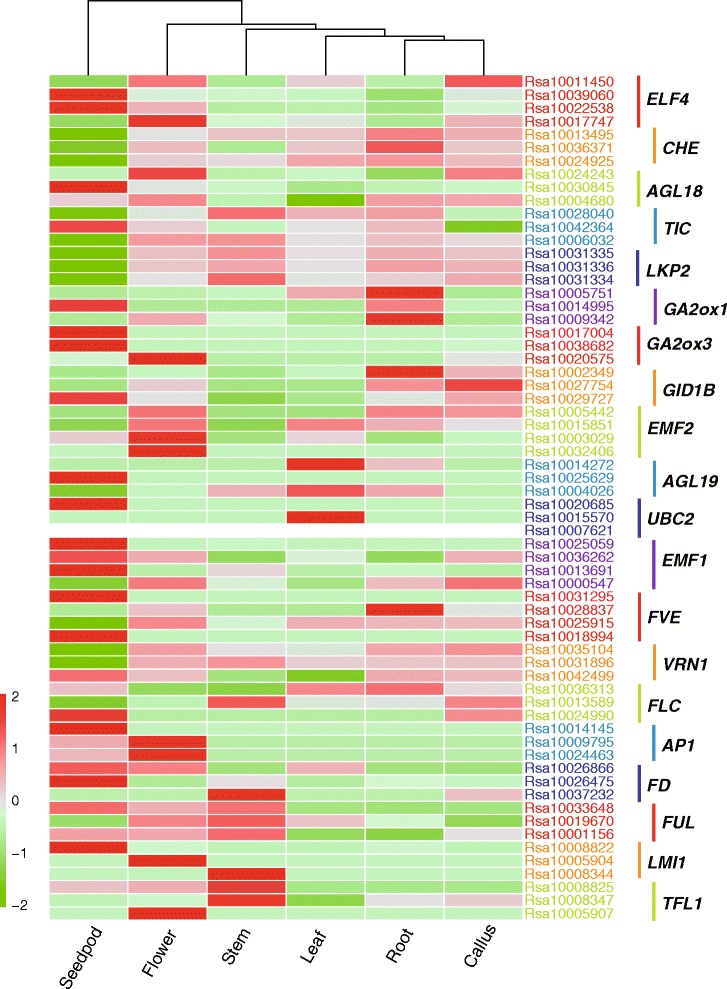
Heatmap of *Raphanus sativus* flowering gene expression profiles. The analyzed tissues are indicated at the bottom of each column. The *R. sativus* gene codes to the right of the expression bar in the same color are homologs of the corresponding genes indicated on the right side with a colored line. The color scale bar at the bottom left of the figure represents log2 transformed FPKM values

Duplicated genes can undergo non-functionalization, neo-functionalization, or sub-functionalization [47]. We chose flowering genes with more than three copies in the *R. sativus* genome to analyze the divergence of the homologous gene expression patterns. Although some genes exhibited similar expression patterns, we also observed considerable differences, suggesting that some homologs are functionally similar, while others are functionally diverse (Fig. 5).

## Discussion

In contrast to the phenotypic effects of vernalization, photoperiod, and GA on *R. sativus* flowering, which have been well studied [48], little is known about the mechanisms mediating the effects. To address this deficiency, we used a bioinformatics approach to analyze the *R. sativus* genes potentially involved in flowering. Based on studies of *A. thaliana* flowering genes, we identified 254 putative *R. sativus* flowering-like genes through a genome-wide comparative analysis. The number of flowering genes in *R. sativus* was slightly less than that in *B. oleracea* and *B. rapa*, which is reasonable considering the genome sizes of the three species [23, 24]. While, homologs for 21 *A. thaliana* flowering genes were not detected in the *R. sativus* genome and many of them have function redundant genes retained in *R. sativus*. The loss of these functionally redundant genes might be due to gene dosage imbalances [49, 50]. However, exactly why certain functionally redundant genes are lost during evolution is unclear. The lost flowering-related genes likely do not affect the core flowering pathways, considering *R. sativus* can still receive various endogenous and environmental cues that facilitate flowering.

Whole genome duplications and triplications are typically followed by a considerable loss of genes. However, the gene dosage hypothesis assumes that genes whose products participate in macromolecular complexes, signaling networks, or transcription in a dose-sensitive manner are over-retained, because an imbalance associated with the loss of one member of a complex or network is likely to decrease fitness [49–51]. Many *B. rapa* circadian clock genes have exhibited preferential retention [25]. However, in this study, we observed that genes related to GA signaling and metabolism were preferentially retained over genes from other pathways. Additionally, genes from the photoperiod pathway, circadian clock, and light signaling gene sets or meristem response and development gene sets were also preferentially retained.

The results of our study suggest that basic flowering pathways are likely relatively conserved between *A. thaliana* and *R. sativus*. Three *R. sativus FLC* homologs were identified in this study, which is consistent with the findings of a transcript-level analysis by Yi et al. (2014), and their functions in transgenic *A. thaliana* have been examined [52]. Jung et al. (2016) also did not detect *R. sativus FRI* homologue through RNA-seq, while we identified two copies and both expressed [45]. The existence and expression of *FLC* and *FRI* in *R. sativus* indicate that the *FLC/FRI* mode of action on vernalization is conserved, as are the components of the autonomous pathway [53]. Previous study reported that the genes in the vernalization pathway are not conserved between dicotyledonous and monocotyledonous plant species [2, 54]. Based on the Ka/Ks ratios, we determined that the sequences of genes related to vernalization pathway were more variable than that of other pathways between *R. sativus* and *A. thaliana*, which indicated vernalization gene sequences also exhibit great sequence diversity among dicotyledon plants. The variation of the vernalization gene sequences may contribute to the rapidly evolutionary capacity in changing thermal requirement to flowering in *R. sativus* [55].

Two *FT* homologs and one copy of *CO* were identified in *R. sativus*. This suggests a *CO-FT* module exists in *R. sativus*, which implies the photoperiod pathway control over flowering evolutionarily conserved in *R. sativus* and *A. thaliana* to a certain extent. In the dark, CO would be efficiently ubiquitinated by the COP1 E3 ligase complex and degraded, which contributed to late flowering in short days [56, 57]. The lost function of *COP1* may suggest that CO would not be degraded in dark in *R. sativus*, which seems to be the reason for that *R. sativus* can flower in both short and long day.

## Conclusions

We identified 254 putative flowering genes during a comparative genome analysis, and classified them into four flowering regulatory pathway gene sets in *R. sativus*. We also comprehensively analyzed the loss, presence, and variation of different pathway genes as well as the expression patterns of the flowering genes in *R. sativus*. Our results reveal that the flowering regulatory network is conserved between *R. sativus* and *A. thaliana* to a certain degree. The flowering-related genes were preferentially retained, especially those associated with GA signaling and metabolism. Furthermore, most of the *R. sativus* flowering genes lost during evolution were functionally redundant, possibly because of gene dosage imbalances. Moreover, analysis of selection pressures indicated that the vernalization and autonomous pathway genes are the most variable in *R. sativus*. Besides, The function loss of *COP1* seems that photoperiod pathway can promote flowering in both short and long day in *R.sativus*.

In summary, our results further systematic and comprehensive understanding of the flowering regulatory molecular networks that evolved after a whole genome triplication event in *R. sativus*, which will be beneficial for breeders aiming to improve and regulate these processes in *R. sativus* and other Brassicaceae species.

## Additional files

Additional file 1: *Raphanus sativus* flowering genes. (XLS 46 kb)

Additional file 2: *Raphanus sativus* flowering genes lost during evolution as well as functionally redundant genes. ‘--’ indicates a lack of functionally redundant genes. (XLS 26 kb)

Additional file 3: Flowering genes identified in *Raphanus sativus*, *Brassica oleracea*, and *Brassica rapa*. (XLS 64 kb)

Additional file 4: The Ka/Ks value of flowering-related genes of *Raphanus sativus* and *Arabidopsis thaliana* in different flowering gene sets. (XLS 54 kb)

Additional file 5: The FPKM (fragments per kilobase of exon per million mapped reads) value of the flowering genes in *Raphanus sativus* flowers, siliques, leaves, stems, callus, and roots. (XLS 70 kb)
